# LB745. Respiratory Syncytial Virus (RSV) Prefusion F Protein Candidate Vaccine (RSVPreF3 OA) is Efficacious in Adults ≥ 60 Years of Age (YOA)

**DOI:** 10.1093/ofid/ofac492.1868

**Published:** 2022-12-15

**Authors:** Michael G Ison, Alberto Papi, Joanne M Langley, Dong-Gun Lee, Isabel Leroux-Roels, Federico Martinon-Torres, Tino F Schwarz, Richard N Van Zyl-Smit, Nancy Dezutter, Nathalie De Schrevel, Laurence Fissette, Marie-Pierre David, Marie Van Der Wielen, Lusine Kostanyan, Veronica Hulstrøm

**Affiliations:** Northwestern University Feinberg School of Medicine, Chicago, Illinois, USA, Chicago, IL; University of Ferrara, St. Anna University Hospital, Ferrara, Italy, Ferrara, Emilia-Romagna, Italy; Dalhousie University, IWK Health and Nova Scotia Health, Halifax, Canada, Halifax, Nova Scotia, Canada; The Catholic University of Korea, Seoul, South Korea, Seoul, Seoul-t'ukpyolsi, Republic of Korea; Ghent University and Ghent University Hospital, Ghent, Belgium, Ghent, Oost-Vlaanderen, Belgium; Hospital Clínico Universitario de Santiago, Santiago de Compostela, Spain, Santiago de Compostela, Galicia, Spain; Klinikum Würzburg Mitte, Campus Juliusspital, Würzburg, Germany, Wuerzburg, Bayern, Germany; University of Cape Town and Groote Schuur Hospital, Cape Town, South Africa, Cape Town, Western Cape, South Africa; GSK, Wavre, Belgium, Wavre, Brabant Wallon, Belgium; GSK, Rixensart, Belgium, Rixensart, Brabant Wallon, Belgium; GSK, Wavre, Belgium, Wavre, Brabant Wallon, Belgium; GSK, Wavre, Belgium, Wavre, Brabant Wallon, Belgium; GSK, Wavre, Belgium, Wavre, Brabant Wallon, Belgium; GSK, Wavre, Belgium, Wavre, Brabant Wallon, Belgium; GSK, Wavre, Belgium, Wavre, Brabant Wallon, Belgium

## Abstract

**Background:**

RSV-associated acute respiratory infections (ARI), particularly lower respiratory tract diseases (LRTD), present a significant disease burden in older adults. Currently, there are no approved vaccines against RSV. We present results from an ongoing study designed to demonstrate the vaccine efficacy (VE) of the AS01_E_-adjuvanted RSVPreF3 OA in adults ≥ 60 YOA.

**Methods:**

This ongoing, phase 3, observer-blind, placebo-controlled, multi-country study (NCT04886596) enrolled adults ≥ 60 YOA from the northern and southern hemispheres. Participants were randomized (1:1) to receive a single dose of RSVPreF3 OA or placebo before the RSV season. The primary objective was to demonstrate VE of a single dose of RSVPreF3 OA in preventing RSV-confirmed LRTD during one RSV season (criterion: lower limit of VE confidence interval [CI] > 20%). VE is reported also against severe RSV-confirmed LRTD, RSV-confirmed ARI, RSV-confirmed LRTD and RSV-confirmed ARI by RSV subtype (RSV-A and RSV-B), and RSV-confirmed LRTD by age, baseline comorbidity and frailty status. RSV-A/B was confirmed by quantitative RT-PCR.

**Results:**

A total of 26,664 participants were enrolled, of whom 24,966 (RSVPreF3 OA: 12,467; placebo: 12,499) were included in the exposed set and 24,960 (RSVPreF3 OA: 12,466; placebo: 12,494) in the efficacy analysis. The mean age was 69.5 (±6.5) years and 51.7% were women. Over a median follow-up of 6.7 months (maximum 10.1 months), 47 RSV-confirmed LRTD episodes were reported (RSVPreF3 OA: 7; placebo: 40), resulting in a VE of 82.6% (96.95% CI: 57.9–94.1), thus the primary objective was met. Consistently high VE across the clinical spectrum of RSV disease, from RSV-confirmed ARI (71.7% [95% CI: 56.2–82.3]) to severe RSV-confirmed LRTD (94.1% [95% CI: 62.4–99.9]) was observed. High VE was seen in different age groups and regardless of RSV subtype, baseline comorbidity or pre-frail status (**Figure 1**). Cumulative incidence curves for RSV-confirmed LRTD and RSV-confirmed ARI showed persistent efficacy throughout the follow-up (**Figure 2**).

**Figure:**
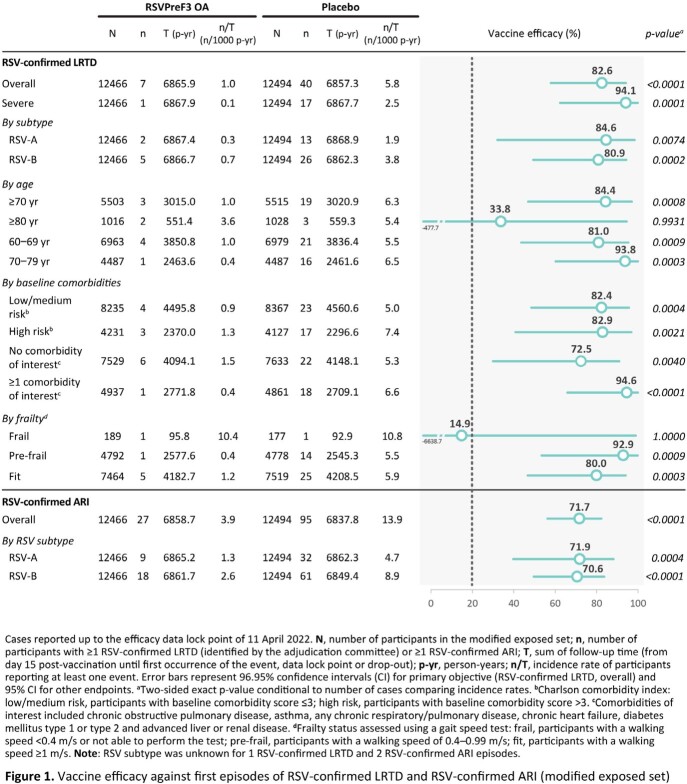

**Figure:**
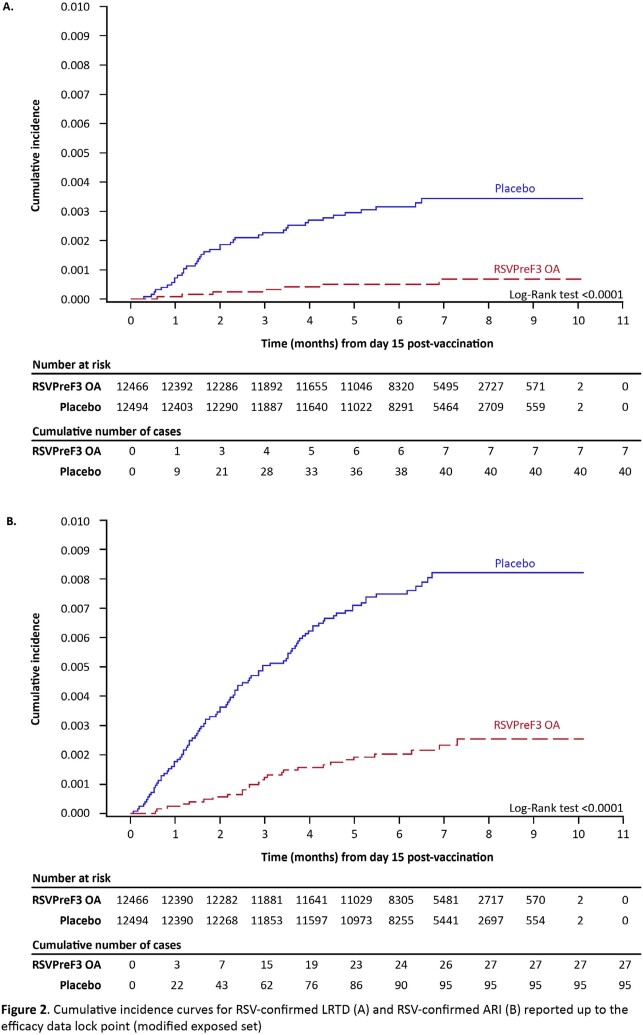

**Conclusion:**

A single RSVPreF3 OA dose is highly efficacious against RSV-confirmed LRTD and RSV-confirmed ARI in adults ≥ 60 YOA, regardless of RSV disease severity, RSV subtype, baseline comorbidity and pre-frail status.

**Funding**: GlaxoSmithKline Biologicals SA.

Abstract and information is also available at the following link : Efficacy results for GSK's older adult RSV vaccine (investis.com).

**Disclosures:**

**Michael G. Ison, MD MS**, GlaxoSmithKline: Advisor/Consultant|GlaxoSmithKline: Grant/Research Support **Alberto Papi, MD**, CHIESI, ASTRAZENECA, GSK, BI, MENARINI, NOVARTIS, ZAMBON, MUNDIPHARMA, SANOFI, AVILLION: Honoraria|CHIESI, ASTRAZENECA, GSK, NOVARTIS, SANOFI, IQVIA, AVILLION, ELPEN PHARMACEUTICALS: Advisor/Consultant|CHIESI, ASTRAZENECA, GSK, NOVARTIS, SANOFI, IQVIA, AVILLION, ELPEN PHARMACEUTICALS: Board Member|CHIESI, ASTRAZENECA, GSK, SANOFI: Grant/Research Support **Joanne M. Langley, MD**, GSK, Merck, Medicago, Sanofi, VBI, VIDO, Entos, Pfizer: Grant/Research Support **Isabel Leroux-Roels, PhD MD**, Curevac: payment to my institution for the conduct of clinical trials|GSK: payment to my institution for the conduct of clinical trials|ICON Genetics: payment to my institution for the conduct of clinical trials|Janssen Vaccines (J&J): Board Member|Janssen Vaccines (J&J): payment to my institution for the conduct of clinical trials|Osivax: payment to my institution for the conduct of clinical trials **Federico Martinon-Torres, MD, PhD, Assoc. Prof**, GlaxoSmithKline, Pfizer, Sanofi, Merck, Moderna, Astra Zeneca, Biofabri, Janssen, Novavax: Advisor/Consultant|GlaxoSmithKline, Pfizer, Sanofi, Merck, Moderna, Astra Zeneca, Biofabri, Janssen, Novavax: Grant/Research Support|GlaxoSmithKline, Pfizer, Sanofi, Merck, Moderna, Astra Zeneca, Biofabri, Janssen, Novavax: Honoraria|GlaxoSmithKline, Pfizer, Sanofi, Merck, Moderna, Astra Zeneca, Biofabri, Janssen, Novavax: Clínical trials fees paid to my institution **Tino F. Schwarz, Prof. Dr. MD**, GlaxoSmithKline: Advisor/Consultant **Richard N. Van Zyl-Smit, PhD MD**, MSD, Pfizer, GSK, Astra Zeneca, Roche, Novartis, Boehringer Ingelheim, Cipla, J&J, Glenmark: Advisor/Consultant|MSD, Pfizer, GSK, Astra Zeneca, Roche, Novartis, Boehringer Ingelheim, Cipla, J&J, Glenmark: Honoraria **Nancy Dezutter, PhD, PharmD**, GlaxoSmithKline: GSK employee|GlaxoSmithKline: Stocks/Bonds **Nathalie De Schrevel, PhD**, GlaxoSmithKline: GSK employee **Laurence Fissette, Master in Statistics**, GlaxoSmithKline: GSK employee|GlaxoSmithKline: Stocks/Bonds **Marie-Pierre David, Master in Statistics**, GlaxoSmithKline: GSK employee|GlaxoSmithKline: Stocks/Bonds **Marie Van Der Wielen, MD**, GlaxoSmithKline: GSK employee|GlaxoSmithKline: Stocks/Bonds **Lusine Kostanyan, MD**, GlaxoSmithKline: GSK employee|GlaxoSmithKline: Stocks/Bonds **Veronica Hulstrøm, PhD MD**, GlaxoSmithKline: GSK employee.

